# Hematological biomarkers of systemic inflammation in predicting long-term treatment response of temporomandibular disorders

**DOI:** 10.1186/s12903-024-04862-x

**Published:** 2024-09-16

**Authors:** Il-San Cho, Jung Hwan Jo, Ji Woon Park

**Affiliations:** 1 Department of Oral Medicine, Seoul National University Dental Hospital, 101, Daehak-ro, Jongno-gu, Seoul, 03080 Korea; 2 Department of Oral Medicine and Oral Diagnosis, School of Dentistry, Seoul National University, 101 Daehak-ro, Jongno-gu, Seoul, 03080 Korea; 3 Dental Research Institute, Seoul National University, 101 Daehak-ro, Jongno-gu, Seoul, 03080 Korea

**Keywords:** Temporomandibular disorders, Hematologic biomarkers, Hemoglobin, Inflammation, Prognosis

## Abstract

**Background:**

Chronic systemic inflammation has been proposed as the underlying mechanism of pain chronicity in several pain conditions. In spite of the growing evidence supporting the role of systemic inflammatory markers as a diagnostic tool, their role has not been analyzed in a well-defined group of temporomandibular disorders (TMD) patients until now. This study aimed to investigate the association between various clinical features and comorbidity levels of TMD in relation to hematological markers and seek its association with long-term treatment response.

**Methods:**

Clinical features and hematological indices including those for systemic inflammation were assessed in TMD patients (*n* = 154). Examinations were re-done after 6 months of conservative treatment. Patients were divided into pain improved and unimproved groups based on ≥ 2 numeric rating scale improvement in pain intensity at 6 months for final analysis.

**Results:**

The portion of patients with low lymphocyte-to-monocyte ratio (*p* = 0.026), total protein (*p* = 0.014), hemoglobin (*p* = 0.040), and mean corpuscular hemoglobin concentration (*p* = 0.042) values showed significant differences according to prognosis groups. Low hemoglobin levels were significantly associated with unfavorable response to long-term treatment (β = 1.706, *p* = 0.018). High pre-treatment pain intensity (β=-0.682, *p* < 0.001) and low Graded Chronic Pain Scale (β = 1.620, *p* = 0.002) could predict significant pain improvement with long-term treatment.

**Conclusions:**

Hematologic assessment could be considered in addition to clinical examination to better determine long-term prognosis in TMD patients.

## Background

Temporomandibular disorders (TMD) is a common form of orofacial pain involving the temporomandibular joints (TMJ) and masticatory muscles [1]. It is known to affect 6–12% of the adult population and is more common in women with a peak prevalence around the age of 20–40 years [2, 3]. According to a recent meta-analysis, the overall prevalence of TMD was reported to be somewhat higher as approximately 31% for adults/elderly and 11% for children/adolescents [4]. Conventional conservative treatments for TMD include education, physical therapy, medication, and intraoral appliance therapy while more recent attempts include temporomandibular joint disc regeneration or replacement using tissue engineering [5, 6]. TMD is known as a multifactorial disease with reported causes involving psychosocial aspects and systemic diseases [1, 2, 7]. While symptoms are mild and self-limiting in most patients, a chronic type of TMD may develop with persistent pain and a higher level of comorbidities including psychological, autonomic, and sleep disturbances [8, 9]. Due to the lack of full understanding involving its initiation and progression, the current diagnostic process for TMD is centered on verifying symptoms through patient interviews, muscle and joint palpations, and imaging of associated structures. This leads to symptomatic treatment rather than pathophysiology-driven therapy which also makes it difficult to predict prognosis [10].

Systemic inflammation may occur as a persistent, low-grade, long-lasting, and non-infective type. Exogenous factors which are also well-known confounders of TMD such as chronic stress, unhealthy habits or environmental changes along with endogenous stimuli could contribute to systemic inflammation [11]. Recently, systemic inflammation has been proposed as a causative factor of pain chronicity in several pain conditions such as fibromyalgia and complex regional pain syndrome [12, 13]. A few studies have also suggested the possibility of immune disturbance in TMD patients, although the results are limited due to their cross-sectional study design or small sample size [14, 15]. The critical role of systemic inflammation has been continuously investigated in major health conditions [16, 17]. However, the current literature on systemic inflammation in TMD as a more localized condition and with well-defined patient groups is extremely limited [18]. Active research to locate hematologic biomarkers of systemic inflammation that are closely related to disease activity and mortality has resulted in the investigation of representative markers including neutrophil-to-lymphocyte ratio (NLR), lymphocyte-to-monocyte ratio (LMR), platelet-to-lymphocyte ratio (PLR), and systemic immune-inflammation index (SII) [19–23]. In spite of the growing evidence supporting the possible role of systemic inflammatory markers as a diagnostic tool, their role has not been analyzed in a well-defined group of TMD patients until now.

Therefore, the aim of this study is to investigate the association between various clinical features and comorbidity levels of TMD in relation to hematologic markers of systemic inflammation and seek its association with long-term response to conventional treatment. And also, to explore the value of inflammatory biomarkers as possible diagnostic indices of TMD prognosis.

## Materials and methods

### Subjects

Consecutive patients who visited the Department of Orofacial Pain Clinic of Seoul National University Dental Hospital complaining of TMD related symptoms from March, 2013 to April, 2019 were studied. TMD was diagnosed following the research diagnostic criteria for TMD (RDC/TMD) [24, 25]. All physical examinations were done by a single experienced orofacial pain specialist who was calibrated by RDC/TMD consortium criteria (*n* = 607).

Exclusion criteria included those with other pain disorders within 6 months of study initiation, uncontrolled hypertension, hyperlipidemia, diabetes mellitus, musculoskeletal, psychiatric, rheumatologic and immune diseases, extended medication use within 2 months of study initiation, recent trauma history or orthognathic surgery, and presence of active inflammation or infection. Rheumatoid factor (RF) and/or antinuclear antibody (ANA) positive patients were referred to a rheumatologist and only included when not diagnosed with a definitive autoimmune and rheumatologic disease (*n* = 163). For longitudinal analysis, patients missing RDC/TMD axis II (*n* = 52) and 6 months follow-up data (*n* = 238) were also excluded. The final group subjected to analysis included 154 patients (mean age 30.2 ± 10.6 years).

All methods were performed in accordance with the Declaration of Helsinki and relevant guidelines. This work was approved by the Institutional Review Board of Seoul National University Dental Hospital (ERI19024). Waiver of additional informed consent was granted considering the retrospective nature of the study and no patient images were utilized in this study.

### Assessment of temporomandibular disorders and related comorbidities

History taking of medical conditions and comorbidities was conducted along with a comprehensive intraoral examination. Degenerative joint disease of the TMJ was diagnosed based on plain radiographs when erosion of the cortical bone, osteophyte, and subcortical cyst formation was observed.

Clinical parameters including comfortable (CMO) and maximum mouth opening (MMO), pain on palpation of muscles (masticatory and cervical) and TMJ capsule area, and pain on mouth opening were examined. Subjective pain intensity was evaluated on a numeric rating scale (NRS, 0–10).

Psychological status and disability levels were evaluated with Symptom Checklist-90-Revision (SCL90R) [26] and RDC/TMD axis II questionnaires [27].

### Hematologic assessment

Samples were taken from the antecubital vein at the first visit before treatment initiation. Complete blood cell counts with white blood cell (WBC) differential, red blood cell (RBC) indices, blood chemistry along with C-reactive protein (CRP), ANA (titers ≥ 1:40 considered positive), and RF were evaluated.

Inflammatory biomarkers including NLR (neutrophil/lymphocyte count), derived NLR (dNLR, absolute neutrophil/[white blood cell-absolute neutrophil count]), LMR (lymphocyte/monocyte count), PLR (platelet/lymphocyte count) and SII (platelet x [neutrophil /lymphocyte count]) were calculated [28, 29]. Cutoff values for NLR (male: 1.634, female 1.662), LMR (male: 5.048, female: 5.598), and PLR (male: 122.726, female: 142.759) were based on mean values from Koreans [30].

### Assessment of long-term treatment response

Conservative treatment included control of contributing factors, self-exercise, occlusal stabilization splint, physical therapy (moist hot pack, ultrasound, electrical stimulation, and low-level laser), and medications including non-steroidal anti-inflammatory drugs. Patients were re-evaluated for CMO, MMO, pain on palpation of masticatory muscles and TMJ capsule, and pain intensity at 6 months from the first examination by the same clinician. For final analysis, patients were differentiated into pain improved and unimproved groups with ≥ 2 NRS improvement in pain intensity as criterion.

### Statistical analysis

Normality of data was tested with Kolmogorov-Smirnov test and methods were selected accordingly. Differences between improved and unimproved groups were analyzed with student’s t-test or Mann-Whitney U test and chi-square test or Fisher’s exact test. Changes in clinical signs at 6 months were analyzed with Wilcoxon singed rank test and McNemar test. Logistic regression analysis was performed to evaluate baseline parameters affecting post-treatment pain improvement. Correlations of each dimension of TMD clinical and hematological parameters were analyzed by Spearman’s correlation coefficients. Receiver operating characteristic (ROC) curve and area under the curve (AUC) were analyzed to evaluate the power of pre-treatment hematological biomarkers in predicting post-treatment pain improvement. All statistical analyses were performed using SPSS 26.0 software (IBM, Chicago, IL, USA). Level of statistical significance was set at *p* < 0.05.

## Results

### Clinical characteristics according to prognosis groups

#### Baseline clinical characteristics

After 6 months of conservative treatment, 107 patients (69.5%) showed significant pain improvement (≥ 2 NRS improvement in pain intensity) and 47 (30.5%) did not.

As shown in Table 1, the pain improved group had a significantly higher pre-treatment pain intensity compared to the unimproved group based on both characteristic pain intensity (*p* = 0.034) and NRS scores on the initial visit (*p* < 0.001). Also, the pain improved group showed more pre-treatment functional disturbance as significantly smaller CMO values (*p* = 0.031) and more patients reporting pain on mouth opening (*p* = 0.011). There were no significant differences in baseline characteristics between the groups regarding confounders including age and gender, RDC/TMD axis I diagnoses, pain origin, and psychological conditions.

**Table 1:** Baseline clinical characteristics according to long-term prognosis groups

Variable	Improved (*n* = 107)	Unimproved (*n* = 47)	Total (*n* = 154)	*P*-value
Age (years)^a^	30.4 (23.0, 36.0)	29.8 (22.0, 37.0)	30.2 (23.0, 36.0)	0.508
Gender (M/F)^b^	(14/93)	(4/43)	(18/136)	0.416
Pain duration (months)^a^	27.0 (1.5, 36.0)	23.1 (3.0, 36.0)	25.8 (2.0, 36.0)	0.610
RDC/TMD axis I diagnosis				
Myofascial pain^b^	72/107 (67.3%)	25/47 (53.2%)	97/154 (63.0%)	0.095
Disc displacement^b^	94/107 (87.9%)	36/47 (76.6%)	130/154 (84.4%)	0.076
Arthralgia/osteoarthritis/osteoarthrosis^b^	87/107 (81.3%)	33/47 (70.2%)	120/154 (77.9%)	0.126
Characteristic Pain Intensity on initial visit (0-100)^a^	45.5 (30.0, 63.0)	38.0 (20.0, 53.3)	43.2 (30.0, 60.0)	0.034^*^
Score ≥ 50^b^	53/107 (49.5%)	16/47 (34.0%)	69/154 (44.8%)	0.075
Disability days^a^	34.9 (0.0, 30.0)	44.0 (0.0, 60.0)	37.7 (0.0, 41.3)	0.262
Days ≥ 90^b^	18/107 (16.8%)	10/47 (21.3%)	28/154 (18.2%)	0.509
GCPS^b^				0.413
Low disability	60/107 (56.1%)	23/47 (48.9%)	83/154 (53.9%)	
High disability	47/107 (43.9%)	24/47 (51.1%)	71/154 (46.1%)	
RDC-DEP^a^	0.712 (0.100, 1.152)	0.717 (0.138, 1.100)	0.714 (0.100, 1.150)	0.928
RDC-DEP ≥ 0.535^b^	54/106 (50.9%)	25/46 (54.3%)	79/152 (52.0%)	0.700
RDC-SOM^a^	0.779 (0.250, 1.250)	0.662 (0.233, 1.080)	0.743 (0.250, 1.165)	0.531
RDC-SOM ≥ 0.500^b^	62/106 (58.5%)	26/46 (56.5%)	88/152 (57.9%)	0.821
RDC-PSOM^a^	0.613 (0.140, 1.000)	0.545 (0.000, 0.893)	0.592 (0.140, 0.965)	0.615
RDC-PSOM ≥ 0.428^b^	56/106 (52.8%)	23/46 (50.0%)	79/152 (52.0%)	0.748
SCL-90R (GSI)^a^	42.8 (36.0, 47.0)	42.1 (35.0, 45.3)	42.6 (36.0, 46.0)	0.686
Score ≥ 60^b^	0.7 (0, 0)	0.6 (0, 0)	0.7 (0, 0)	0.995
Pain origin^b^				0.112
Myogenous	34/107 (31.8%)	18/47 (38.3%)	52/154 (33.8%)	
Arthrogenous	5/107 (4.7%)	4/47 (8.5%)	9/154 (5.8%)	
Mixed	51/107 (47.7%)	13/47 (27.7%)	64/154 (41.6%)	
Pain intensity on initial visit (NRS)^a^	5.1 (4.0, 6.0)	3.2 (1.0, 5.0)	4.5 (3.0, 6.0)	< 0.001^*^
CMO (mm)^a^	36.6 (27.0, 45.0)	40.6 (32.0, 49.0)	37.9 (29.0, 47.0)	0.031^*^
MMO (mm)^a^	42.6 (36.0, 50.0)	44.5 (38.0, 50.0)	43.2 (38.0, 50.0)	0.250
Pain on mouth opening^b^	69/107 (64.5%)	20/47 (42.6%)	89/154 (57.8%)	0.011^*^
Pain on capsule palpation^b^	55/107 (51.4%)	18/47 (38.3%)	73/154 (47.4%)	0.134
Pain on masticatory muscle palpation^b^	77/107 (72.0%)	30/47 (63.8%)	107/154 (69.5%)	0.313
Pain on cervical muscle palpation^b^	47/107 (43.9%)	20/47 (42.6%)	67/154 (43.5%)	0.874
Headache^b^	55/107 (51.4%)	24/47 (51.1%)	79/154 (51.3%)	0.969
Sleep disturbance^b^	34/107 (31.8%)	16/47 (34.0%)	50/154 (32.5%)	0.782
Neck and shoulder pain^b^	62/107 (57.9%)	27/47 (57.4%)	89/154 (57.8%)	0.954
Low back pain^b^	46/107 (43.0%)	15/47 (31.9%)	61/154 (39.6%)	0.196
Leg and arm pain^b^	18/107 (16.8%)	5/47 (10.6%)	23/154 (14.9%)	0.321
Gastrointestinal disorder^b^	26/107 (24.3%)	14/46 (30.4%)	40/153 (26.1%)	0.428
DJD on radiograph^b^	66/107 (61.7%)	28/47 (59.6%).	94/154 (61.0%)	0.805

M, male; F, female; RDC/TMD, research diagnostic criteria/temporomandibular disorders; GCPS, Graded Chronic Pain Scale; NRS, numeric rating scale; RDC-DEP, depression score of RDC/TMD axis II; RDC-SOM, somatization score of RDC/TMD axis II; RDC-PSOM, somatization score of RDC/TMD axis II without pain items; SCL-90R, Symptom Checklist 90 Revision; GSI, Global Severity Index; CMO, comfortable mouth opening; MMO, maximum mouth opening; DJD, degenerative joint disease of temporomandibular joint

^a^Mann-Whitney U test: Median (lower quartile, upper quartile)

^b^Chi-square test: number of positive subjects

^*^Significant difference, *P* < 0.05

#### Changes in clinical signs with treatment

As shown in Table 2, all measured clinical and functional indices were significantly improved in the pain improved group with 6 months’ conservative treatment however, only the percentage of those with pain on masticatory muscle palpation significantly decreased in the unimproved group. Functional disturbance and pain persisted in this group.

**Table 2:** Changes in clinical signs with treatment according to long-term prognosis groups

Improved (*n* = 107)							Unimproved (*n* = 47)					
	Pain intensity (NRS)^a^	CMO (mm)^a^	MMO (mm)^a^	Pain on mouth opening^b^	Pain on capsule palpation ^b^	Pain on masticatory muscle palpation ^b^	Pain intensity (NRS)^a^	CMO (mm)^a^	MMO (mm)^a^	Pain on mouth opening^b^	Pain on capsule palpation ^b^	Pain on masticatory muscle palpation^b^
Baseline	5.1 (4.0, 6.0)	36.6 (27.0, 45.0)	42.6 (36.0, 50.0)	69/107 (64.5%)	55/107 (51.4%)	77/107 (72.0%)	3.2 (1.0, 5.0)	40.6 (32.0, 49.0)	44.5 (38.0, 50.0)	20/47 (42.6%)	18/47 (38.3%)	30/47 (63.8%)
6 months	1.2 (0.0, 2.0)	43.1 (38.0, 50.0)	44.7 (45.0, 50.0)	36/107 (33.6%)	34/107 (31.8%)	45/107 (42.1%)	3.3 (1.0, 5.0)	41.7 (36.0, 49.0)	44.0 (38.0, 49.0)	21/47 (44.7%)	15/47 (31.9%)	17/47 (36.2%)
P-value	< 0.001^*^	< 0.001^*^	< 0.001^*^	< 0.001^*^	0.002^*^	< 0.001^*^	0.730	0.296	0.884	1.000	0.607	0.002^*^

NRS, numeric rating scale; CMO, comfortable mouth opening; MMO, maximum mouth opening; DJD, degenerative joint disease of temporomandibular joint

^a^Wilcoxon signed rank test: Median (lower quartile, upper quartile)

^b^McNemar test: number of positive subjects

^*^Significant difference, *P* < 0.05

### Hematologic marker levels according to prognosis groups

As shown in Table 3, significantly more patients in the pain improved group had an abnormally low pre-treatment LMR value (*p* = 0.026). NLR, dNLR, PLR, and SII values were higher and LMR value was lower in the pain improved group although the difference was not statistically significant. Among red blood cell parameters, the pain improved group had a significantly higher hemoglobin (Hgb) concentration (*p* = 0.040) and more patients in the unimproved group had an abnormally low Hgb level (*p* = 0.046). Also, the mean corpuscular hemoglobin concentration (MCHC) was significantly higher in the improved group (*p* = 0.042). Significantly more patients in the pain unimproved group had an abnormally high protein concentration (*p* = 0.014).

**Table 3:** Hematologic markers according to long-term prognosis groups

Variable	Improved (*n* = 107)	Unimproved (*n* = 47)	Total (*n* = 154)	*P*-value
NLR^a^	1.996 (1.391, 2.266)	1.871 (1.310, 2.125)	1.958 (1.348, 2.228)	0.778
NLR group (≥ 1.662 (F), ≥ 1.634 (M))^c^	65/107 (60.7%)	27/47 (57.4%)	92/154 (59.7%)	0.701
dNLR^a^	1.492 (1.049, 1.771)	1.437 (1.016, 1.695)	1.475 (1.044, 1.726)	0.868
LMR^a^	4.528 (3.605, 5.247)	4.913 (3.816, 5.692)	4.646 (3.697, 5.429)	0.139
LMR group (≤ 5.598 (F), ≤ 5.048 (M))^c^	88/107 (82.2%)	31/47 (66.0%)	119/154 (77.3%)	0.026^*^
PLR^a^	143.531 (106.140, 171.552)	130.922 (102.189, 154.289)	139.683 (105.716, 165.983)	0.317
PLR group (≥ 142.759 (F), ≥ 122.726 (M))^c^	51/107 (47.7%)	15/47 (31.9%)	66/154 (42.9%)	0.069
SII (*10^3^/µl)^a^	527.147 (338.204, 596.657)	481.066 (311.107, 581.324)	513.084 (326.638, 594.803)	0.770
WBC (*10^3^/µl)^a^	6.10 (5.01, 6.89)	6.30 (5.44, 6.90)	6.16 (5.09, 6.89)	0.267
WBC group (≥ 10.0*10^3^/µl)^d^	3/107 (2.8%)	0/47 (0.0%)	3/154 (1.9%)	0.553
RBC (*10^6^/µl )^a^	4.52 (4.24, 4.83)	4.46 (4.16, 4.60)	4.50 (4.23, 4.75)	0.165
RBC group (≥ 5.40*10^6^/µl)^d^	2/107 (1.9%)	4/47 (8.5%)	6/154 (3.9%)	0.071
Hgb (g/dL)^a^	13.7 (12.9, 14.5)	13.4 (12.3, 13.8)	13.6 (12.8, 14.4)	0.040^*^
Hgb group (≤ 12.0 g/dL)^d^	5/107 (4.7%)	7/47 (14.9%)	12/154 (7.8%)	0.046^*^
Hct (%)^b^	40.4 (3.0)	39.7 (3.3)	40.2 (3.1)	0.189
Hct group (≤ 36.0%)^d^	6/107 (5.6%)	3/47 (6.4%)	9/154 (5.8%)	1.000
MCV (fL)^a^	89.6 (87.1, 92.2)	89.5 (87.5, 92.5)	89.5 (87.1, 92.2)	0.844
MCV group (≤ 79.0 fL)^d^	0/107 (0.0%)	0/47 (0.0%)	0/154 (0.0%)	
MCH (pg)^b^	30.3 (1.3)	30.0 (1.4)	30.2 (1.4)	0.117
MCH group (≤ 26.0 pg)^d^	0/107 (0.0%)	0/47 (0.0%)	0/154 (0.0%)	
MCHC (g/dL)^a^	33.8 (33.2, 34.5)	33.5 (32.8, 34.1)	33.7 (33.1, 34.4)	0.042^*^
MCHC group (≤ 32.0 g/dL)^d^	0/107 (0.0%)	2/47 (4.3%)	2/154 (1.3%)	0.092
Platelet (*10^3^/µl)^a^	259.2 (210.0, 285.0)	255.0 (229.0, 290.0)	257.9 (213.0, 287.0)	0.702
Platelet group (≥ 400.0*10^3^/µl)^d^	2/107 (1.9%)	0/47 (0.0%)	2/154 (1.3%)	1.000
Total protein (g/dL)^a^	7.6 (7.3, 7.8)	7.7 (7.4, 7.9)	7.6 (7.3, 7.9)	0.218
Total protein group (≥ 8.0 g/dL)^c^	8/107 (7.5%)	10/47 (21.3%)	18/154 (11.7%)	0.014^*^
ESR (mm/hr)^a^	9.0 (3.0, 12.0)	10.0 (5.0, 13.0)	9.3 (3.8, 13.0)	0.089
ESR group (≥ 20.0 mm/hr)^d^	9/107 (8.4%)	3/47 (6.4%)	12/154 (7.8%)	1.000
CRP (mg/dL)^a^	0.09 (0.04, 0.09)	0.11 (0.03, 0.08)	0.10 (0.03, 0.09)	0.441
CRP group (≥ 0.50 mg/dL)^d^	3/99 (3.0%)	2/46 (4.3%)	5/145 (3.4%)	0.652
RF positivity^d^	9/107 (8.4%)	2/47 (4.3%)	11/154 (7.1%)	0.505
FANA positivity^c^	13/106 (12.3%)	6/47 (12.8%)	19/153 (12.4%)	0.931

NLR, neutrophil-to-lymphocyte ratio; dNLR, derived NLR ratio; LMR, lymphocyte-to-monocyte ratio; PLR, platelet-to-lymphocyte ratio; SII, systemic immune-inflammation index; CRP, C-reactive protein; ESR, erythrocyte sedimentation rate; RF, rheumatoid factor; FANA, fluorescent antinuclear antibody; WBC, white blood cell; RBC, red blood cell; Hgb, hemoglobin; Hct, hematocrit; MCV, mean corpuscular volume; MCH, mean corpuscular hemoglobin; MCHC, mean corpuscular hemoglobin concentration

^a^Mann-Whitney U test: Median (lower quartile, upper quartile)

^b^Student’s t-test: mean (SD)

^c^Chi-square test: number of positive subjects

^d^Fisher’s exact test

^*^Significant difference, *P* < 0.05

### Correlation between clinical characteristics and hematologic markers

RBC levels were significantly correlated with 6 months’ post-treatment CMO (*r* = 0.288, *p* < 0.001) and MMO (*r* = 0.257, *p* = 0.001) values. Hgb concentration was significantly correlated with 6 months’ post-treatment CMO (*r* = 0.240, *p* = 0.003) and MMO (*r* = 0.234, *p* = 0.003) values. Hematocrit was significantly correlated with 6 months’ post-treatment CMO (*r* = 0.261, *p* = 0.001) and MMO (*r* = 0.255, *p* = 0.001). Mean corpuscular volume (MCV) was significantly correlated with pain duration (*r*=-0.176, *p* = 0.030). Erythrocyte sedimentation rate (ESR) was significantly correlated with 6 months’ post-treatment CMO (*r*=-0.168, *p* = 0.038) and MMO (*r*=-0.160, *p* = 0.047).

### Clinical and hematological parameters associated with refractory TMD pain

Logistic regression analysis was carried out with pre-treatment clinical and hematologic markers as independent variables and unimproved TMD pain as the dependent variable.

As shown in Table 4, high disability level of the Graded Chronic Pain Scale (GCPS) (β = 1.620, *p* = 0.002), and low pre-treatment pain intensity on NRS (β=-0.682, *p* < 0.001) was associated with long-term refractory TMD pain.

**Table 4:** Baseline clinical characteristics associated with refractory temporomandibular disorders

Variable	Standardized β	Standard error	95% CI	*P*-value	
Age	0.002	0.021	0.962–1.045	0.912	
Gender (1:M, 0:F)	-1.406	0.847	0.047–1.288	0.097	
Pain duration	-0.002	0.007	0.983–1.012	0.740	
GCPS group (1:high disability, 0: low disability)	1.620	0.527	1.799–14.199	0.002^*^	
RDC-DEP	0.599	0.530	0.645–5.142	0.258	
RDC-SOM	-1.016	1.214	0.034–3.909	0.403	
RDC-PSOM	0.114	1.245	0.098–12.869	0.927	
NRS	-0.682	0.149	0.378–0.677	< 0.001^*^	
CMO (mm)	0.068	0.036	0.998–1.149	0.057	
MMO (mm)	-0.021	0.045	0.897–1.069	0.638	
Pain on capsule palpation	0.250	0.511	0.471-3.500	0.625	
Pain on masticatory muscle palpation	-0.004	0.515	0.363–2.734	0.994	
Pain on cervical muscle palpation	-0.236	0.506	0.293–2.131	0.642	

CI, confidence interval; M, male; F, female; GCPS, Graded Chronic Pain Scale; RDC-DEP, depression score of RDC/TMD axis II; RDC-SOM, somatization score of RDC/TMD axis II; RDC-PSOM, somatization score of RDC/TMD axis II without pain items; NRS, numeric rating scale; CMO, comfortable mouth opening; MMO, maximum mouth opening

Given values were obtained by logistic regression analysis

^*^Significant difference, *P* < 0.05

As shown in Table 5, abnormally low Hgb level (β = 1.706, *p* = 0.018) was associated with refractory TMD pain.

**Table 5:** Baseline hematologic markers associated with refractory temporomandibular disorders

Predictor variable	Standardized β	Standard error	95% CI	*P*-value	
NLR group (≥ 1.662 (F), ≥ 1.634 (M))	0.431	0.441	0.649–3.651	0.328	
LMR group (≤ 5.598 (F), ≤ 5.048 (M))	-0.909	0.467	0.166–1.006	0.052	
PLR group (≥ 142.759 (F), ≥ 122.726 (M))	-0.787	0.433	0.195–1.063	0.069	
WBC group (≥ 10.0*10^3^/µl)	-19.614	21032.766	0.000-.	0.999	
RBC group (≥ 5.40*10^6^/µl)	1.648	1.020	0.704–38.410	0.106	
Hgb group (≤ 12.0 g/dL)	1.706	0.723	1.335–22.707	0.018^*^	
Platelet group (≥ 400.0*10^3^/µl)	-18.371	25347.989	0.000-.	0.999	
ESR group (≥ 20.0 mm/hr)	-0.084	0.816	0.186–4.549	0.918	
CRP group (≥ 0.50 mg/dL)	0.278	1.072	0.161–10.792	0.796	
RF positive	-0.685	0.889	0.088–2.879	0.441	
FANA positive	0.315	0.569	0.449–4.182	0.580	

CI, confidence interval; NLR, neutrophil-to-lymphocyte ratio; LMR, lymphocyte-to-monocyte ratio; PLR, platelet-to-lymphocyte ratio; ESR, erythrocyte sedimentation rate; CRP, C-reactive protein; WBC, white blood cell; RBC, red blood cell; Hgb, hemoglobin; RF, rheumatoid factor; FANA, fluorescent antinuclear antibody

Groups were defined as 0: normal, 1: abnormal

^*^Significant difference, *P* < 0.05

### Effectiveness of pre-treatment hematologic markers in predicting long-term refractory TMD pain

**Fig. 1:**
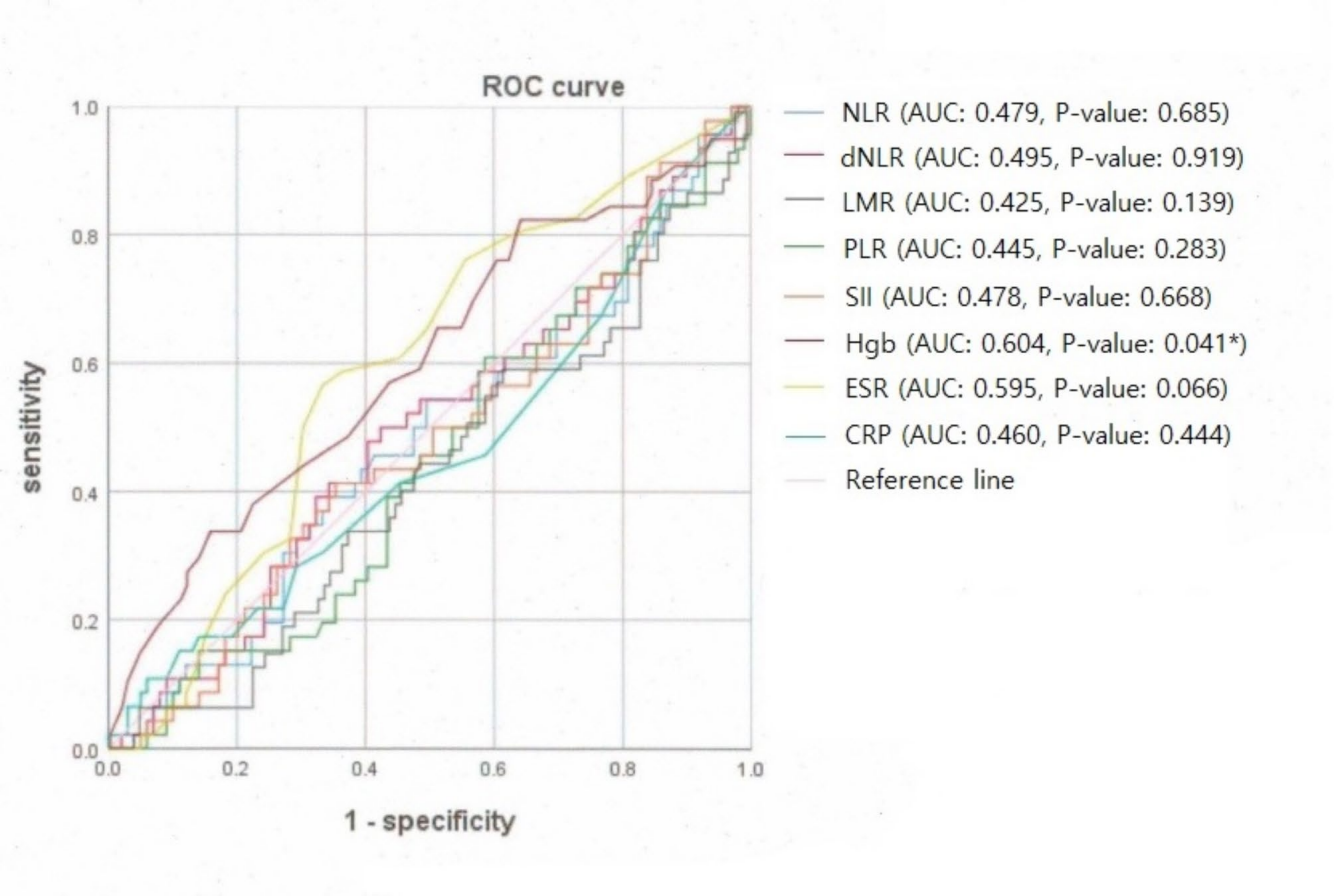
Receiver operating characteristic (ROC) curves for long-term refractory TMD pain. The diagnostic ability was significant for hemoglobin concentration. AUC, area under the curve; NLR, neutrophil-to-lymphocyte ratio; dNLR, derived NLR ratio; LMR, lymphocyte-to-monocyte ratio; PLR, platelet-to-lymphocyte ratio; SII, systemic immune-inflammation index; Hgb, hemoglobin; ESR, erythrocyte sedimentation rate; CRP, C-reactive protein

As shown in Table 6; Fig. 1, receiver operating characteristic (ROC) curve analyses showed that Hgb had sufficient predictive power to discriminate refractory TMD pain with a cutoff of 13.2 g/dL (area under the curve [AUC] = 0.604, *p* = 0.041).

**Table 6:** Sensitivity, specificity, PPV, NPV, and error rate of baseline hematologic markers in evaluating refractory temporomandibular disorders

		Pain group		AUC	Cutoff value	Sensitivity (%) [95% CI]	Specificity (%) [95% CI]	PPV (%) [95% CI]	NPV (%) [95% CI]	Error rate (%)	*P*-value
		Improved	Unimproved								
NLR	< 1.761	54	21	0.479	1.761	55.3 [40.1, 69.8]	50.5 [40.6, 60.3]	32.9 [26.3, 40.3]	72.0 [64.0, 78.8]	48.1	0.685
	≥ 1.761	53	26								
dNLR	< 1.421	60	24	0.495	1.421	48.9 [34.1, 63.9]	56.1 [46.2, 65.7]	32.9 [25.4, 41.3]	71.4 [64.3, 77.6]	46.1	0.919
	≥ 1.421	47	23								
LMR	> 4.340	55	26	0.425	4.340	44.7 [30.2, 59.9]	51.4 [41.5, 61.2]	28.8 [21.8, 37.0]	67.9 [60.7, 74.4]	50.7	0.139
	≤ 4.340	52	21								
PLR	< 122.589	45	18	0.445	122.589	61.7 [46.4, 75.5]	42.1 [32.6, 52.0]	31.9 [26.2, 38.2]	71.4 [62.0, 79.3]	52.0	0.283
	≥ 122.589	62	29								
SII (*10^3^/µl)	< 518.267	70	28	0.478	518.267	40.4	65.4	33.9	71.4	42.2	0.668
	≥ 518.267	37	19			[26.4, 55.7]	[55.6, 74.4]	[25.0, 44.2]	[65.6, 76.7]		
Hgb (g/dL)	> 13.2	60	20	0.604	13.2	57.5	56.1	36.5	75.0	43.5	0.041*
	≤ 13.2	47	27			[42.2, 71.7]	[46.2, 65.7]	[29.3, 44.3]	[67.4, 81.3]		
ESR (mm/hr)	< 9.5 ≥ 9.5	71 36	21 26	0.595	9.5	55.3 [40.1, 69.8]	66.4 [56.6, 75.2]	41.9 [33.3, 51.1]	77.2 [70.5, 82.7]	37.0	0.066
CRP (mg/dL)	< 0.06 ≥ 0.06	54 45	27 19	0.460	0.06	41.3 [27.0, 56.8]	54.6 [44.2, 64.6]	29.7 [22.0, 38.8]	66.7 [59.7, 73.0]	49.7	0.444

NLR, neutrophil-to-lymphocyte ratio; dNLR, derived NLR ratio; LMR, lymphocyte-to-monocyte ratio; PLR, platelet-to-lymphocyte ratio; SII, systemic immune-inflammation index; Hgb, hemoglobin; ESR, erythrocyte sedimentation rate; CRP, C-reactive protein

Cutoff value was determined by Euclidean Method, Sensitivity was obtained from TP/(TP + FN) x 100, Specificity was obtained from TN/(TN + FP) x 100, PPV was obtained from TP/(TP + FP) x 100, NPV was obtained from TN/(TN + FN) x 100, Error rate was obtained from (FN + FP)/(TN + TP + FN + FP)

## Discussion

The results of this study showed that low Hgb levels were associated with long-term refractory TMD pain. Also, less patients had a low LMR value which is known to reflect an enhanced inflammatory status. Additionally, the total protein concentration reached abnormally high levels more frequently in refractory TMD patients. Such results point towards the possible involvement of systemic inflammation in TMD patients that do not respond well to conventional treatment.

This is the first study to investigate the relationship between clinical characteristics and hematological biomarkers of systemic inflammation such as NLR, dNLR, LMR, PLR, and SII in TMD patients. The growing interest in research to better understand disease states or predict prognosis with simple blood testing has led to the investigation of biomarkers involving nonspecific inflammation. And previous studies support the role of such indices in evaluating disease severity [16–23]. When TMD patients were grouped according to long-term treatment response, less patients in the unimproved group showed lower LMR values (*p* = 0.026) and the mean value was higher compared to the improved group although the difference did not reach statistical significance. Such a finding could be considered more significant since the applied cutoff value was based on data derived from Koreans and differentially applied according to gender. Studies show that mean values of hematologic markers of systemic inflammation including LMR, may differ according to ethnic group and gender hence, applying a cutoff value derived from other races could affect results [30]. However, this point was often not considered in other studies. Lower LMR values are known to reflect a higher degree of systemic inflammation and is frequently associated with poor prognosis. On the other hand, there is one research on breast cancer patients showing that low LMR has been reported as a predictive factor of favorable response [31]. In this aspect, the results of our study may appear contradictory to previous studies reporting a negative relationship between LMR and the level of postoperative pain [32]. Also, studies on rheumatoid arthritis investigating the relationship between pain levels and the same markers of systemic inflammation as in this study generally report that higher levels of inflammation are related to more pain [21]. Unfortunately, it is difficult to directly compare the results from such studies since most were of a cross-sectional design and smaller sample size which limits their validity to support a causal relationship. Since the decrease in pain intensity with TMD treatment was greater when the pre-treatment pain intensity was higher in our patients, it could be indirectly interpreted that the presence of nonspecific inflammation pre-treatment could be linked to higher pain intensity and better prognosis in terms of long-term pain improvement. From a different perspective, such a result could suggest that TMD with a larger inflammatory component responds more favorably to conventional treatment. Acute inflammation generally responds well to treatment in the majority of cases while, chronic pain is more often associated with low-grade inflammation [33]. The cutoff values of inflammatory indices used in this study were derived from healthy adults and data on values from specific disease groups is rare. This may have contributed to the failure of other hematologic markers of inflammation in reaching statistical significance and underlines the need to produce appropriate cutoff values that are associated with specific disease characteristics. Another point to consider is the cause of the change in inflammatory marker levels. Since those with active inflammation were excluded, it could be said that the inflammation present in the TMD patients is of a nonspecific nature which may have affected long-term treatment response. But still it is difficult to differentiate between systemic and local inflammation based on the indices investigated in this study.

Hgb levels in those with refractory TMD pain were decreased in this study. Also, abnormally low Hgb level was associated with unfavorable treatment response based on logistic regression analysis. Hgb level may also be considered as an indicator of inflammatory conditions. Anemia of chronic disease is most commonly present in infectious, inflammatory or neoplastic diseases. Mediators of the inflammatory response are considered to be involved in the development of anemia [34]. Low Hgb levels were associated with higher disease activity, structural damage, and joint dysfunction in rheumatoid arthritis (RA) [35]. Also, Hgb reflected inflammatory status and disease activity in systemic lupus erythematosus, so could be used as a marker to predict treatment outcome [36]. The pathogenesis of anemia in chronic diseases involves abnormalities of iron absorption, release from macrophages, and dysfunction of cytokine networks, all of which can result in inadequate erythropoiesis [35]. It has been known that the production of cytokines in RA leads to a decrease in iron availability and plays a direct toxic effect on erythropoietin. In RA patients, the increased activation of inflammatory cells causing excessive cytokine production including tumor necrosis factor (TNF)-α, interleukin (IL)-1β, and IL-6 acts on erythropoietin progenitor cells, promoting hemolysis and causing subsequent reduction in the number of circulating red blood cells [35]. Inflammation could lead to the development of inflammatory anemia. Lower levels of Hgb are found in autoimmune hemolytic anemia which is accompanied by systemic inflammation [37]. Based on such studies reporting that increased levels of inflammatory cytokines could result in low levels of Hgb, this could indirectly suggest the possibility of increased inflammatory cytokine levels in our refractory TMD patients. A recent study found that TMD patients with high disability showed increased inflammatory cytokine levels including IL-β, -6, -10, and TNF-α [15]. Another study showed that IL-8 and IgG levels were significantly increased in the high disability TMD group [14]. Inflammation in the nervous system play significant roles in many chronic pain conditions. Certain cytokines/chemokines may directly activate nociceptive sensory neurons initiating and maintaining pathologic pain. Certain inflammatory cytokines are also involved in central sensitization and resulting hyperalgesia/allodynia [38].

Another finding of this study related to the low Hgb level of those with refractory TMD pain was the lower MCHC level in the unimproved group compared to the improved group. MCHC reflects the amount of hemoglobin responsible for oxygen transportation in the RBCs and is related to iron storage. A lower MCHC level was associated with poor prognosis in chronic heart disease patients and could be applied as a biomarker for evaluating the prognosis of chronic obstructive pulmonary disease, both which are accompanied by increased systemic inflammation [39].

In our study, more patients of the unimproved group showed abnormally increased total protein levels. Total protein levels increase in inflammatory states as chronic inflammation is associated with substantial changes in protein metabolism [40]. Whole body protein synthesis and breakdown is increased in inflammatory conditions such as inflammatory bowel disease [41]. Also, increased levels of total protein are found in other diseases including multiple myeloma which are known to have an inflammatory component in its pathogenesis [42]. A previous review reported that vitamin D deficiency and TMD were associated [43] while vitamin D has been implicated in the underlying mechanism of inflammation and insulin resistance [44]. Such literature additionally supports the role of sub-inflammation in TMD and the need to investigate the matter in relation to already known substance of TMD etiology including vitamins and hormones. However, the current existing literature on systemic inflammation in TMD is more focused on analyzing cytokine levels from venous blood [14, 15, 18] and future studies should include a wider range of systemic inflammatory indices to provide a comprehensive view on the issue.

As for clinical variables related to unfavorable TMD prognosis, lower pre-treatment pain intensity showed significant association. The higher the initial subjective pain level, the greater the long-term decrease with treatment. This result is in line with a study based on TMD showing that higher pain intensity and more widespread pain pre-treatment were significantly related to more pain improvement with treatment [45].

On the other hand, high disability level based on GCPS was significantly associated with long-term refractory TMD pain. GCPS reflects not only the intensity of pain but also the level of disturbance in daily activities that the patient perceives to have. Interference in activities is also caused by common comorbidities of TMD such as psychological and sleep disturbances in addition to the pain intensity itself [15, 46]. Depression is a well-known contributing factor of chronic TMD as is primary sleep disorders such as obstructive sleep apnea and insomnia [47–49].

There are several limitations of this study to be considered. First, this study was a single-center study of a retrospective nature. Although known confounders of both TMD and hematologic markers were controlled, this was based on information from medical records and additional verification of systemic conditions was not carried out. Exclusion of subjects based on the presence of certain systemic conditions including other pain disorders was done by medical history taking through a structured interview and this could have resulted in the inclusion of certain data which was inappropriate for analysis. Secondly, hematologic information was not collected at the long-term follow-up point limiting the direct analysis between clinical and hematologic values after 6-months’ treatment. Future studies should involve collection of both data sets to allow a more precise establishment of their interrelationship. However, the aim of this study was to tentatively evaluate the possibility of hematologic indices as prognostic parameters and the longitudinal aspect of our study fulfills such an objective. Thirdly, patient diagnosis of this study followed the RDC/TMD since DC/TMD had not yet been implemented in the clinic during the designated study period. Results based on the more recent DC/TMD diagnosis could differ and future studies should apply the most recent diagnostic criteria for up-to-date information [50]. Fourthly, there may be statistical bias since a large number of patients were excluded from the parent population due to the lack of long-term follow-up data. Finally, our study did not produce results based on different gender and age groups although both factors were matched when comparing the groups. Also, all data was from Koreans so the results may not universally apply to other ethnic groups. The hematologic biomarkers investigated in this study are known to be affected by various factors including age, gender, race, and adverse health habits so, future studies should be designed to consider such conditions as well-designed prospective research to further validate the role of inflammatory biomarkers in the diagnosis of TMD.

In conclusion, high LMR values were observed in refractory TMD patients. Also, low Hgb and high total protein levels were associated with poor long-term prognosis in TMD with conventional treatment. Such results could indicate the possible role of nonspecific inflammation in chronic TMD pathogenesis. Their validity for clinical usage should be further evaluated in addition to clinical factors such as pain intensity and GCPS in TMD.

## Data Availability

The raw data supporting this study are not in the public domain but are available upon reasonable request from the corresponding author.

## Abbreviations

TMD: Temporomandibular disorders
TMJ: Temporomandibular joints
NLR: Neutrophil-to-lymphocyte ratio
LMR: Lymphocyte-to-monocyte ratio
PLR: Platelet-to-lymphocyte ratio
SII: Systemic immune-inflammation index
RDC/TMD: Research diagnostic criteria for TMD
RF: Rheumatoid factor
ANA: Antinuclear antibody
CMO: Comfortable mouth opening
MMO: Maximum mouth opening
NRS: Numeric rating scale
WBC: White blood cell
RBC: Red blood cell
Hgb: Hemoglobin
MCHC: Mean corpuscular hemoglobin concentration
MCV: Mean corpuscular volume
CRP: C-reactive protein
ESR: Erythrocyte sedimentation rate
ROC: Receiver operating characteristic
AUC: Area under the curve
GCPS: Graded chronic pain scale
TNF: Tumor necrosis factor
IL: Interleukin
